# A cross‐sectional study of distress: A cancer response

**DOI:** 10.1002/nop2.460

**Published:** 2020-04-01

**Authors:** Hanna Ekman, Alexandra Pettersson, Liselotte Jakobsson, Pernilla Garmy

**Affiliations:** ^1^ Department of Oncology Kristianstad Central Hospital Kristianstad Sweden; ^2^ Department of Oncology Lund University Hospital Lund Sweden; ^3^ Department of Health Sciences Kristianstad University Kristianstad Sweden

**Keywords:** cancer, distress, distress thermometer, fatigue, financial, oncology, psychosocial, rehabilitation, treatment, working age

## Abstract

**Aim:**

To describe the experience of distress in people with cancer of working age.

**Design:**

A cross‐sectional study.

**Methods:**

In this cross‐sectional study, patients (*N* = 168) with both ongoing (*N* = 56) and completed treatment (*N* = 105) completed the Distress Thermometer and the detailed problem list. Data were analysed by descriptive and analytical statistics.

**Results:**

A large proportion of patients (29%) continued to experience high distress (>3 according to the Distress Thermometer) even after treatment was completed. Patients experienced several problems after treatment had ended such as fatigue (44%), sleep problems (34%), worries (31%), pain (31%), tingling in hands and feet (31%) and problems with memory/concentration (30%). Patients with financial/insurance problems had significantly higher distress than those who did not have these problems.

## 1 INTRODUCTION

The number of cancer cases is increasing continuously, this means that one out of three Swedes will be diagnosed with cancer during their lifetime. If this trend continues, every second Swede will suffer from a cancer diagnosis by 2030 (Swedish Cancer Society, 2017).

All people with cancer experience some degree of distress as a direct result of the disease, regardless of which phase of the disease the patient is in. Cancer‐related distress can include feelings about vulnerability, grief and fear, as well as problems that can be disabling, such as depression, anxiety, panic, social isolation and existential and spiritual crises. Distress occurs from the time of the diagnosis and might affect a patient's everyday life for a long time to come (NCCN, 2013). Furthermore, distress can lead to poorer quality of life, poorer adherence to treatment and affect the patient's satisfaction with medical care (Tonsing & Vungkhanching, 2018). Many people with cancer are of working age and will return to work after treatment and sick leave. It is important that their cancer care is suitable for the various complications that might arise during the various phases of cancer and its treatments. This can also explain how living with a cancer diagnosis affects patients.

## 2 BACKGROUND

There are large variations in the degree of distress experienced by patients and the areas that affect distress including gender, age and marital status. Research shows that more women than men experience a high degree of distress (Mehnert et al., 2016). In general, younger patients have a higher degree of distress regardless of gender, whereas younger women experience higher distress compared with older women (Jorgensen, Laursen, Garne, Sherman, & Sogaard, 2016). Men differ from women in that it is the middle‐aged men who 4 have the highest distress, rather than younger men, while the older men have the lowest distress. There is also a variation between those who are married and young and middle‐aged who experience higher distress than those who are older. Furthermore, middle‐aged single persons have a higher degree of distress than younger and older people (Burgoyne et al., 2015). For women, the areas that contribute to distress are psychosocial problems, such as changed self‐image, family‐related problems and sexual problems. Men more commonly have problems of an existential nature (Koyama et al., 2016).

The Distress Thermometer is an assessment tool recommended for the assessment of cancer rehabilitation needs both nationally and internationally (NCCN, 2013). There is no clear consensus on the threshold score of the Distress Thermometer at which a patient should be considered unable to cope and requiring further intervention. On the one hand the optimal cut‐off value for people with cancer is generally considered to be 4, while a cut‐off value of 6 is considered acceptable in patients with active treatment. A cut‐off value of 6 is also considered appropriate for patients who are in a palliative phase of their illness (Ma et al., 2014). On the other hand, it is considered that the optimum cut‐off value is 7, where the significantly higher value is considered to ensure that it is patients who experience a high 5 degree of distress referred to more specialized measures based on their needs (Bulli, Miccinesi, Maruelli, Katz, & Paci, 2009). It is important that assessments with the Distress Thermometer are made regularly during treatment and follow‐up. According to healthcare professionals, the Distress Thermometer is best applied in the middle of active treatment or a short time after, whereas patients believe that the Distress Thermometer is optimal from the middle of the treatment but even better after the treatment is completed when symptoms and consequences are more prominent (Biddle et al., 2016). Based on a meta‐analysis with 42 studies and 14,808 patients, it was concluded that the Distress Thermometer is a validated instrument for detecting potential distress in people with cancer (Ma et al., 2014). A Swedish study including patients with different cancer diagnoses showed the same result (Thalén‐Lindström, Larsson, Hellbom, Glimelius, & Johansson, 2013). The use of the Distress Thermometer—which is relatively short—is an effective way of assessing the degree of distress experienced by the patient and the problems that contribute to the experience of distress (Bulli et al., 2009). Thayssen et al. (2016) examined how patients experience completing the Distress Thermometer before a doctors' visit and propose that this stimulates the patient's ability to reflect on their situation and gives them the opportunity to address what they want from their doctor or nurse. The patients consider it positive and valuable to complete the Distress Thermometer.

Many published studies have been conducted in non‐European settings, about distress and its effects on people with cancer, using the Distress Thermometer (Burgoyne et al., 2015; Koyama et al., 2016; Tonsing & Vungkhanching, 2018), some studies have also been conducted in Europe (Biddle et al., 2016; Jorgensen et al., 2016) but only a few in Sweden (Thalén‐Lindström et al., 2013), and none of the studies have had focused on working‐age patients; therefore, it is important to illuminate how distress is manifested in people with cancer of working age in a European country such as Sweden. Due to the consequences of treatment/illness, the target group is at risk for an extended reduced working capacity with accompanying financial worry. Thus, the aim of this study is to describe this distressing experience in working‐age people with cancer in southern Sweden via the Distress Thermometer.

## 3 DESIGN

This cross‐sectional study is drawing on empirical material from another study, which aimed to investigate the cancer rehabilitation experiences of working‐age cancer survivors; therefore, a more detailed description of the data collection and the participants is found in Garmy and Jakobsson (2018).

### 3.1 Method

The study was performed at the Cancer Rehabilitation Clinic on a medium size hospital in southern Sweden. The Cancer Rehabilitation Clinic cooperated with the Swedish Social Insurance Agency in five neighbouring municipalities with between 7000–85,000 inhabitants per municipality. A questionnaire and information were distributed in October 2016 via mail to all patients over the age of 18 years who had a cancer diagnosis and were enrolled in the Swedish Social Insurance Agency in the included municipalities from January 2013–April 2016 (*N* = 384). One reminder was sent after 4 weeks. Written informed consent was requested and was completed by hand and returned together with the questionnaire in a provided stamped reply envelope. A total of 168 patients answered the questionnaire (response rate of 44%).

### 3.2 Survey questionnaire

#### 3.2.1 Background data

The questionnaire consisted of questions about background data: gender, age, civil state, children, birth country, other diseases, ongoing treatment, long‐term treatment, psychiatric disease and addictions.

#### 3.2.2 The distress thermometer

The Distress Thermometer is an assessment tool that measures distress and is provided by the National Comprehensive Cancer Network (NCCN). The form contains two parts: the thermometer where the patient estimates the degree of distress in the last week on a visual analog scale from 0–10 and a problem list with 39 common problem areas related to cancer and its treatment (Eckerdal, 2017). By understanding the underlying causes of distress, the health professional can better apply targeted interventions and thus reduce the distress of the people with cancer (Tonsing & Vungkhanching, 2018).

### 3.3 Analysis

Data were analysed by descriptive and analytical statistics with means, standard deviations and percentages. A *t* test was used to investigate if there were age or gender differences between those who had ongoing treatment versus those who had completed treatment. Bivariate analyses were used with a chi‐squared test between those who had ongoing and completed treatment in relation to the issues of the Distress Thermometer. Bivariate analysis and multiple logistic regression were used to compare the 0–3 and 4–10 distress groups and identify risk factors for distress. The variables for the risk factors associated with distress agreed with practical experience. The Hosmer Lemeshow and the Nagelkerke *R* ^2^ tests were reported for the logistic regression analysis. IBM SPSS version 22 was used for analysis.

## 4 RESULTS

A total of 168 persons reported on having distress or not, and 161 persons reported on ongoing or completed cancer treatment. Most (65%) of the participants completed their cancer treatment while 35% had ongoing cancer treatment in some form; however, the groups did not differ based on gender, country of birth and family situation (Table 1).

**Table 1:** Description of the sample (*N* = 161)

Sample description	Ongoing treatment (*N* = 56, 35%)	Completed treatment (*N* = 105, 65%)	*p*‐value
Age in years (means, *SD*)	56.18 (8.6 *SD*)	56.90 (9.2 *SD*)	.634^a^
Sex			.926^b^
Women, *N* (%)	38 (67.8%)	72 (68.5%)	
Men, *N* (%)	18 (32.2%)	33 (31.5%)	
Country of birth			.165^b^
Sweden, *N* (%)	50 (91%)	101 (96%)	
Nordic countries, *N* (%)	2 (3.6%)	0 (0%)	
Europe, *N* (%)	3 (5.4%)	3 (3%)	
Outside Europe, *N* (%)	0 (0%)	1 (1%)	
Living with a partner, *N* (%)	48 (89%)	78 (76%)	.061^b^
Parent of children, *N* (%)	50 (91%)	94 (91%)	.719^b^
Children under the age of 18, *N* (%)	15 (30%)	19 (20%)	.400^b^

Note

Participants were divided based on whether they had ongoing treatment or had completed cancer treatment.

Missing cases: 0%–7%.

Abbreviation: *SD*, standard deviation.

a *t* test.

b Chi‐squared test.

A high degree of distress was defined as >3 according to the Distress Thermometer. Table 2 shows the prevalence of various problem areas that affected distress and the stated degree among those with ongoing or completed treatment. Of those who had completed their treatment, 29% still had a high degree of distress. Of those with ongoing treatment, 48% had a high degree of distress. Patients experienced several problems after treatment had ended such as fatigue (44%), sleep problems (34%), worries (31%), pain (31%), tingling in hands and feet (31%) and problems with memory/concentration (30%). Patients with financial/insurance problems had significantly higher distress than those who did not have these problems.

**Table 2:** Description of the Distress Thermometer (*N* = 161) where the participants were divided based on whether they had an ongoing or completed cancer treatment

Distress Thermometer	Ongoing treatment (*N* = 56)	Completed treatment (*N* = 105)	*p*‐value^a^
Distress 0–3, *N* (%)	29 (52%)	75 (71%)	**.013**
Distress 4–10, *N* (%)	27 (48%)	30 (29%)	
Practical problems			
Child care, *N* (%)	0 (0%)	1 (1%)	.464
Housing, *N* (%)	3 (5%)	3 (3%)	.425
Insurance/financial, *N* (%)	9 (16%)	9 (9%)	.150
Transportation, *N* (%)	4 (7%)	4 (4%)	.354
Work/School, *N* (%)	10 (18%)	13 (12%)	.344
Treatment decisions, *N* (%)	8 (14%)	5 (5%)	**.035**
Family problems			
Dealing with children, *N* (%)	3 (5%)	4 (4%)	.647
Dealing with partner, *N* (%)	5 (9%)	6 (6%)	.441
Ability to have children, *N* (%)	2 (4%)	1 (1%)	.242
Family health issues, *N* (%)	18 (32%)	21 (20%)	.087
Emotional problems			
Depression, *N* (%)	9 (16%)	8 (8%)	.096
Fear, *N* (%)	18 (32%)	11 (10.5%)	**.001**
Nervousness, *N* (%)	13 (23%)	16 (15%)	.210
Sadness, *N* (%)	24 (43%)	23 (22%)	**.005**
Worry, *N* (%)	35 (62.5%)	33 (31%)	**<.001**
Loss of interest in usual activities, *N* (%)	13 (23%)	15 (14%)	.155
Spiritual/ religious concerns, *N* (%)	4 (7%)	2 (2%)	.095
Physical problems			
Appearance, *N* (%)	8 (14%)	9 (9%)	.261
Bathing dressing, *N* (%)	4 (7%)	1 (1%)	**.031**
Breathing, *N* (%)	11 (20%)	2 (2%)	**.000**
Changes in urination, *N* (%)	7 (12.5%)	5 (5%)	.075
Constipation, *N* (%)	8 (14%)	8 (8%)	.178
Diarrhoea, *N* (%)	10 (18%)	6 (6%)	**.014**
Eating, *N* (%)	5 (9%)	8 (8%)	.771
Fatigue, *N* (%)	38 (68%)	46 (44%)	**.004**
Feeling swollen, *N* (%)	19 (34%)	14 (13%)	**.002**
Fevers, *N* (%)	2 (4%)	4 (4%)	.939
Getting around, *N* (%)	20 (36%)	23 (22%)	.059
Indigestion, *N* (%)	7 (12.5%)	4 (4%)	**.037**
Memory/concentration, *N* (%)	25 (45%)	31 (30%)	.055
Mouth sores *N* (%)	5 (9%)	6 (6%)	.441
Nausea, *N* (%)	12 (21%)	5 (5%)	**.001**
Nose dry/ congested, *N* (%)	12 (21%)	13 (13%)	.254
Pain, *N* (%)	20 (36%)	32 (30.5%)	.498
Sexual, *N* (%)	11 (20%)	16 (15%)	.476
Skin dry/itchy, *N* (%)	23 (41%)	21 (20%)	**.004**
Sleep, *N* (%)	20 (36%)	36 (34%)	.856
Substance use, *N* (%)	0 (0%)	0 (0%)	
Tingling in hands and feet, *N* (%)	25 (45%)	32 (30.5%)	.073

Note

*p*‐values < .05 were considered significant and marked in bold.

Missing cases: 0%.

a Chi‐squared test.

The degree of distress was dichotomized into groups of low (0–3) and high (4–10) distress. The bivariate analysis explored whether there was any significant correlation between the degree of distress and age, sex, living with partner or alone, having children under 18 years old, living in the home, other bodily diseases, mental illness, completed treatment, ongoing treatment or some form of long‐term treatment. The analysis shows that there was a significant relationship (*p* < .05) between insurance/finance problems and a high degree of distress. There was also a significant relationship (*p* < .05) between the degree of distress and cancer treatment (Table 3).

**Table 3:** Bivariate analysis of the risk factors linked to the high degree of distress ≥ 4 (*N* = 168)

Variables	Distress 0–3 (*N* = 110)	Distress 4–10 (*N* = 58)	*p*‐value^a^
Age under: 40	4 (4%)	3 (5.4%)	.085
Age: 40–60	53 (50%)	28 (50%)	
Age over: 60	49 (46.2%)	25 (45%)	
Sex: men	38 (35%)	17 (30%)	.470
Sex: women	71 (65%)	41 (70%)	
Living with partner	84 (80%)	45 (82%)	.784
Living alone	21 (20%)	10 (18%)	
Children under 18 living home	18 (19%)	16 (29%)	.168
Insurance/finance	6 (6%)	13 (22%)	**.001**
Have or have had other physical diseases	40 (38%)	25 (45%)	.423
Have or have had mental illness	5 (5%)	4 (6.9%)	.532
Have completed the cancer treatment	75 (71%)	30 (29%)	**.013**
Have ongoing cancer treatment	29 (52%)	27 (48%)	
Have any long‐term treatment	37 (35%)	32 (57%)	**.006**

Note

*p*‐values < .05 were considered significant and are marked in bold.

Missing cases: 0%–9.5%.

a Chi‐squared test.

The multiple regression analysis describes whether there was any significant relationship between distress ≥ 4 and factors such as age, sex, living with partner or alone, having children under 18 years old, insurance/finance, other bodily diseases, mental illness, completed treatment and whether they underwent any form of long‐term treatment. The multiple logistic regression analysis showed that there was a significant relationship (*p* < .05) between the degree of distress and insurance/finance problems. Ongoing or completed treatment was not a significant factor in this analysis (Table 4).

**Table 4:** Multiple logistic regression analysis of the risk factors linked to the high degree of distress ≥ 4 (*N* = 144)

Variables	OR	95% CIs for OR	*p*‐value
Age	1.007	0.953–1.064	.811
Sex	1.227	0.520–2.893	.641
Living with a partner or living alone	1.297	0.474–3.547	.613
Children under 18 living home	1.225	0.651–2.306	.530
Insurance/ finance	4.953	1.312–18.694	**.018**
Have or have had other bodily diseases	1.116	0.498–2.502	.789
Have or have had a mental illness	0.648	0.143–2.937	.573
Have completed the cancer treatment	1.517	0.630–3.653	.353
Have any long‐term treatment	0.612	0.265–1.411	.249

Note

Hosmer and Lemeshow Test, *p* = .442; Nagelkerke *R* ^2^ = .121.

*p*‐values < .05 were considered significant and are marked in bold.

Abbreviations: CI, confidence intervals; OR, odds ratio.

## 5 DISCUSSION

The patients in our study described a high degree of fatigue regardless of whether they were undergoing treatment or if they had finished treatment. Kurt and Unsar (2010) demonstrated that symptoms such as fatigue are common during treatment but could also persist for as long as years after the end of treatment. A meta‐analysis of fatigue reported that patients who completed their treatment during the first half of the year experienced a progressively decreasing degree of fatigue, but it could take up to several years before it completely disappeared (Kurt & Unsar, 2010). According to Islam et al. (2014), fatigue can be a factor that is directly associated with difficulties in eventually returning to work. For this reason, it is very important to identify those patients who have a continued high degree of fatigue even after treatment has been completed. Thus, they can have an individually adapted rehabilitation plan for successful recovery.

Previous work showed that patients who have completed their treatment are expected to quickly and fully return to their everyday life as if nothing has happened (Stanton et al., 2005). However, our findings suggest that these expectations are unreasonable because several patients still exhibited fatigue, pain and insomnia despite the completion of treatment. Rather, the result can be understood within the context of the transition from one life phase to another. For example, this might involve changed health conditions, changed relationships or other expectations based on ability. The transition consists of three phases: entry, passage and exit. During the entry phase, a major life change occurs and is either positively or negatively conditioned—a passage that continues can be affected by the environment and can be more difficult or easier. The final phase of the transition is the exit, which means that the individual has reached a phase of personal development and greater stability (Meleis & Trangenstein, 1994). This means that the patient, after completion of treatment, now has a new “normal” because it is no longer possible to return to the state before the cancer due to continuing symptoms. Thus, there is a long‐term and continuing need for rehabilitation in this patient group.

Despite this need, Stanton et al. (2005) reported that individuals who completed their treatment no longer receive the same amount of active support and thus feel less secure. Willems et al. (2016) claimed that people with cancer still have unmet needs that are directly related to their disease and treatment after termination of treatment. These needs are not adequately addressed by healthcare professionals. The results of our study imply that it is important to pay attention to the rehabilitation needs even after treatment has finished—especially given that the population is getting older and the age limit for pensions rises. This is relevant because the working age increases, and more people of working age will have cancer.

Distress during treatment usually causes worse conditions for managing disease and treatment. Distress can also markedly influence life after treatment, including difficulties in returning to day‐to‐day work or longer sick leaves. Therefore, there are longer social and personal impacts. Therefore, it is important for cancer care to identify patients who have a higher degree of distress leading to an increased rehabilitation need, that is support and guidance from oncology specialists. The best possible conditions are to return to a normal life and work with less strain on the healthcare system and society. Further research is needed to identify a structure that provides knowledge on how these interventions are best performed.

### 5.1 Study limitations

One strength of this method is that the Distress Thermometer is a validated instrument that is user‐friendly. In addition, the participants had previously been in contact with the Distress Thermometer and used the tool together with healthcare personnel, which significantly reduces the risk of misunderstandings. The study was cross‐sectional, which means that it captures a snapshot of the conditions prevailing at a specific time. Thus, our study cannot address how the conditions change over a longer period.

The response rate was 44% after the reminder. We actively decided not to distribute a second reminder because the participants had cancer and might be preoccupied. We cannot exclude the possibility that the low participation rate might have been due to the intended participants having progressed in their disease with little strength for participation; they might also have not received the questionnaire because they may have also moved or have been hospitalized. The low participation might also affect the generalizability of the result. This is a sensitive subject, and it is unclear if the dropout needs to be high for cancer studies.

### 5.2 Conclusion

This study showed that distress is a common problem regardless of whether people are undergoing cancer treatment or have completed treatment. This distress can have an impact on their work life, which is increasingly relevant due to older retirement ages and should be studied from a social perspective. From the oncology perspective, these results increase our understanding of which patients are at risk of experiencing a higher degree of distress and thus have poorer conditions for compliance with oncological treatment. It also explains the larger perspective including the risk of lower quality of life. In addition to the patients’ benefits, the data can also have a socio‐economic benefit because many known consequences of cancer treatment can be prevented if they are identified early. This will reduce the burden on the healthcare system.

## CONFLICT OF INTEREST

No conflict of interest.

## ETHICAL APPROVAL

This study was approved by the Regional Ethical Review Board in Lund, Sweden, EPN, (2016/424) and followed the principles of the Helsinki. Before the study started, written informed consent was collected from the participants. It was clarified that the survey was voluntary and participation could be withdrawn.

## Data Availability

Data are available on request due to privacy/ethical restrictions.
